# Consumption and Charity

**Published:** 1912-12-14

**Authors:** 


### CONSUMPTION AND CHARITY.

The present consumption crusade appeals chiefly for funds wherewith to teach the people the proper method of combating tuberculosis. That much good may be done in that way is true, but it would be a mistake to give anyone the impression that when once the right management of such cases has been told to sufferers and their families the duty of the charitable public will be at an end. When all the teaching has been explained, and even—a longer and more difficult matter—when all the teaching has been assimilated, there will still be need of sanatoria, and there will still be need of charity.

We cannot at once and by mere preaching put our people into a good state of health. In the first place, a hatred of fresh air seems to be innate among the poor. With houses smaller, less well ventilated, and less favourably situated than those of their richer neighbours, they cherish a profound antipathy to every breath of air that they might have. They have a morbid fear of draughts, by which term they designate every perceptible breeze that enters a house, and sometimes seem to have that imaginative sensitiveness which enabled the man in the story to catch cold under a plate-glass window which he mistakenly believed to be open. It is not easy to account for the habit of overloading themselves with clothes. If one could hope that a term of sanatorium treatment would induce them to realise that the skin needs air, it would be money well invested, from a public health point of view, to make every head of a family receive it. But it is doubtful if the instruction would be welcomed.

Nor can we hope that the conditions of all labour will become wholly sanitary for a long time to come. Much has been done in factories, mines, and all places where large numbers of workers are employed, to render the conditions healthy, but in small shops and in establishments where many assistants sleep in one room, often by no means as large or airy as is desirable, and also where home work is done, we may expect tuberculosis to persist for a long time yet. Consumption may be checked and lessened, but it is still far from being extirpated.

The conditions of cure include several items, but of almost equal importance are good treatment until the disease is arrested, and good conditions to which to return—and the latter is often the more unattainable of the two. One might add early treatment, but in the case of the classes which depend for the week's maintenance on the week's earnings, a consumptive will struggle along until he can work no longer, and this not only lessens his own chances but tends to injure the health of his family. The study of a number of representative cases * of consumptive working-men, treated either at the Mundesley Sanatorium or in the open-air wards of Sheffield Infirmary, and gathered together by Dr. Noel Dean Bardswell, show clearly that, if an effective and permanent cure is to be hoped for, three things are necessary—prolonged sanatorium treatment; when that is done with, work of a not too exhausting kind in healthy conditions; and a sufficiency of good and nourishing food. Where these conditions could all be obtained the disease was completely arrested; where they were not present the results were often unfavourable. Something depends on the intelligence and fidelity of the patient, shown by his carrying out at home, to the best of his ability, the methods and treatment he received at the sanatorium; but much also depends on the conditions to which he has to return. Those who have no one dependent upon them have the best chance, for they can, if need be, take work that is less well paid if it is healthier or less severe. But a man with a family looking to him for support is tempted to take up whatever work he can get, and to consider money wages more than suitable conditions. Moreover, if a man is to remain contentedly in a sanatorium for a sufficiently long time to profit by the treatment, he must feel assured that his wife and children are not suffering through his absence. Where either private or organised charity undertook the care of the family, and where it was so extended as to enable the patient to get suitable work and wait until he could obtain it, the results were invariably favourable. When the patient had to go back to the old bad conditions, they were as invariably bad.

The great merit of Dr. Bardswell's reports lies in the fact that they were carried on for so long a time —from three to six years in the cases that recovered, and until death in the others. Some of the latter are pathetic—of a patient trying his best to get suitable employment, and being driven back at last by necessity to unhealthy or over-strenuous conditions. Take, for example, a youth of eighteen, who was discharged after twenty-eight weeks' treatment. He was a hawker, and his work was healthy enough; but he had a widowed mother and several brothers and sisters who were entirely dependent on his earnings. The earnings were variable, and his home conditions were bad. The last time he was visited "he was found bedridden in a dirty room which was extremely stuffy, every window being closed, and the cracks between the sashes covered with sandbags. The patient, with his mother and the rest of the family, were on the parish, and were living in great poverty." Dr. Bardswell's comment is: "This patient's record, though very disappointing and to some extent discouraging, has its value, for it shows what a hopeless matter it is to benefit permanently the consumptive poorer classes, if they must needs return to conditions of bad hygiene, inadequate food and general poverty. . . . Financial assistance to his family and suitable employment, viz., one at which he could earn a living wage, might well have turned the scale in the patient's favour."

Set against this the case of another youth, aged eighteen a grocer's assistant, who after sixteen weeks of treatment left the Sheffield Infirmary with his lung disease "very considerably arrested," but not wholly cured. Through the kindness of a former employer he was enabled to emigrate to Australia,

* *The Consumptive Working-Man: What Can Sanatoria do for Him.* By Noel Dean Bardswell, M.D., M.R.C.P., F.R.S. Edin., Medical Superintendent, King Edward VII. Sanatorium. London: The Scientific Press.

where he led rather a roving life but managed to maintain his health. This patient left the Infirmary in 1899, and his last report, in February 1906, was still satisfactory. He was then in New Zealand and still at work. For him sanatorium treatment had been a great success. The moral hardly needs to be told. The one youth had to return to irregular work, and his earnings had to support others than himself. The second seems indeed to have taken up irregular work, but in a better climate than ours, and with no one but himself to consider. If a place did not suit him, he could go elsewhere; if he found one kind of work unhealthy he was free to try another. Given equal advantages, each case might have resulted in cure; it was their after conditions that determined their fate.

Let us add to these cases two cases of married men. One, a sawyer, aged 36, had a wife and two young children. After 14 weeks' treatment at Mundesley, he returned to his home at Walworth. During his stay at Mundesley the Charity Organisation Society had been allowing him 23s. per week, which paid the fee (17s. per week) for his treatment, and left something over for the maintenance of his family, who were also receiving 10s. a week from a benefit club. He left the sanatorium in May 1903, and in February 1906 was still well and at work. In this case the help of the Charity Organisation Society made it possible for the patient to take the prolonged treatment necessary, and they also interested themselves in him after his discharge. But for this assistance the result might have been different. Against this set the case of a box-maker, aged 33, a widower with three children. Leaving Sheffield Infirmary after thirteen weeks' treatment, he got a job as driver of a van, at something like ten shillings a week less than his former wages. But the work was light and was out of doors. Unfortunately he exchanged it for more remunerative work in a bakehouse, with the result that within six months his disease reappeared and he died. In this case the need—real or imaginary—of earning more money led to the patient undertaking work for which he had not the requisite health.

